# Effects of dietary glutathione supplementation on the growth performance, nutrient digestibility, antioxidant function and intestinal morphology in growing male minks

**DOI:** 10.1371/journal.pone.0342801

**Published:** 2026-02-18

**Authors:** Beibei Zhang, Yang Ci, Keru Li, Yanhong Xu, Menghao Li, Wenli Li

**Affiliations:** 1 College of Animal Science and Technology, Qingdao Agricultural University, Qingdao, China; 2 Qingdao Phagepharm Biotechnology Co., Ltd, Qingdao, China; 3 Tuanwang Animal Husbandry and Veterinary Station, Yantai, China; University of Illinois, UNITED STATES OF AMERICA

## Abstract

This study investigated the effects of dietary glutathione (GSH) supplementation on growth performance, nutrient digestibility, amino acid digestibility, antioxidant capacity, and intestinal morphology in growing male minks. Sixty two-month-old male minks were divided into 6 groups and fed diets supplemented with 0 (control), 50, 100, 150, 200 and 250 mg/kg GSH over a 9-week trial. The results showed numerically higher growth performance and pelt quality in the groups receiving 50–200 mg/kg GSH, but these differences were not statistically significant (*P* > 0.05). Dietary supplementation with 150–250 mg/kg GSH significantly increased crude fat digestibility, while 100–200 mg/kg GSH enhanced cysteine digestibility and 150 mg/kg GSH elevated the digestibility of isoleucine, leucine, tyrosine, and phenylalanine during the first four weeks (*P* < 0.05). At the end of the experiment, total serum superoxide dismutase (T-SOD) activity was significantly increased with 150–250 mg/kg GSH (*P* < 0.05). Serum glutathione peroxidase activity was significantly elevated by 150–200 mg/kg GSH (*P* < 0.05). Hepatic T-SOD activity was significantly increased by 100 and 200 mg/kg GSH (*P* < 0.05), and glutathione transferase activity was upregulated by 50, 100, and 200 mg/kg GSH (*P* < 0.05). Additionally, 250 mg/kg GSH significantly raised hepatic GSH level (*P* < 0.05), and both 100 and 250 mg/kg GSH significantly elevated the hepatic GSH/GSSG ratio (*P* < 0.05). Furthermore, 200 mg/kg GSH significantly increased the villus height in the duodenum and jejunum (*P* < 0.05). In conclusion, dietary GSH supplementation increased nutrient digestibility, antioxidant capacity and intestinal health in growing minks, with 200 mg/kg showing greater benefits, thereby providing valuable guidance for its practical use in mink nutrition.

## Introduction

L-Glutathione (GSH) is a natural tripeptide composed of glutamate, cysteine, and glycine, containing a sulfhydryl group [1]. As the most abundant non-protein thiol compound in living organisms, GSH is widely distributed in the cells of mammals, plants, and microorganisms, playing a crucial role in defense against oxidative stress [2–4]. GSH interacts directly with reactive oxygen species (ROS) and serves as a cofactor for glutathione peroxidase (GSH-Px) in detoxifying hydrogen peroxide and hydroperoxides [5]. GSH possesses various physiological functions, including antioxidant activity, detoxification, regulation of cell proliferation and apoptosis, immune modulation, and participation in nutrient metabolism [6,7]. Liang et al. [8] found that dietary GSH supplementation improved growth performance and intestinal barrier function by enhancing antioxidant capacity and mitochondrial function in weaned piglets under oxidative stress. In addition, Wang et al. [9] demonstrated that GSH supplementation in low-fishmeal diets had no significant effects on growth performance or whole-body composition but improved antioxidant capacity and intestinal morphology in shrimp.

The mink (*Neovison vison*) is a valuable fur-bearing carnivorous mammal, whose pelt quality is the primary determinant of its economic value. The synthesis of keratin, the primary protein of fur, is highly dependent on the availability of sulfur-containing amino acids like cysteine [10]. As GSH contains cysteine in its structure, dietary GSH supplementation may serve as both an antioxidant and a potential source of bioavailable cysteine, which could theoretically support keratin synthesis and fur quality. Although the antioxidant role of GSH has been confirmed in many species [8,9,11], its efficacy as a dietary supplement is often species-specific due to differences in physiology and metabolism. To our knowledge, there are few data on the protective effect of dietary GSH supplementation in minks. Thus, this study was designed to evaluate the effects of dietary supplementation with different levels of GSH on growth performance, pelt quality, nutrient digestibility, serum and hepatic antioxidant capacity, and intestinal morphology in growing minks.

## Materials and methods

### Experiment design

The experiment was approved by the Animal Ethics Committee of the College of Animal Science and Technology, Qingdao Agricultural University (Approval No. DKY20200601). A total of 60 two-month-old weaned male minks of the short-haired black variety with similar body weights were randomly divided into 6 groups, with 10 replicates (cages) per group and one mink per replicate. The control group received a basal diet, and experimental groups were fed the basal diet supplemented with 50, 100, 150, 200, or 250 mg/kg GSH. The basal diet was formulated based on T/SDAA 0095–2024 [12] requirements and commercial recommendations. The composition and nutritional levels of the basal diet are shown in Table 1. The trial consisted of a 1-week adaptation period followed by a 9-week experimental period. GSH (99.0% purity) was purchased from Guangzhou Shengda Biotechnology Co., Ltd.

**Table 1:** Ingredient composition (as-fed basis) and nutrient levels (dry-matter basis) of basal diets.

Items (%, otherwise indicated)	Weeks 1–4	Weeks 5–9
Ingredients		
Duck skeleton	18	18
Egg	15	10
Chicken intestine	15	15
Chicken skeleton	5	5
Anglerfish head	10	10
Mixed marine fish	10	10
Cod bone	10	10
Extruded corn	9.5	9.5
Extruded soybean	2	2
Porcine hemoglobin powder	1.5	1.5
Fish meal	1	1
Soybean oil	0	5
Premix^a^	3	3
Total	100	100
Nutrient levels^b^		
Dry matter	38.47	37.48
Metabolizable energy (MJ/kg)	16.45	17.81
Gross energy (MJ/kg)	19.99	21.23
Crude protein	36.60	31.57
Crude fat	17.86	23.73
Ash	12.04	10.67
Calcium	2.90	2.57
Total phosphorus	1.06	0.91

^a^The premix provided the following per kg of diets: VA 300 000 IU, VB_1_ 670 mg, VB_2_ 300 mg, VB_6_ 300 mg, VB_12_ 3.3 mg, pantothenic acid 400 mg, nicotinic acid 1 300 mg, folic acid 30 mg, biotin 17 mg, VC 4 000 mg, VE 5 000 mg, VK_3_ 30 mg, Fe 2 000 mg, Mn 1 000 mg, Zn 1 700 mg, Cu 170 mg, Se 13 mg, I 17 mg.

^b^The metabolizable energy is calculated value and others are measured values. Metabolizable energy was calculated according to the formula: Metabolizable energy = (0.85 × Crude protein % × 4.5 + 0.90 × Crude fat % × 9.5 + 0.75 × Nitrogen-free extract % × 4.0) × 4.184; Nitrogen-free extract % = 1-Crude protein %-Crude fat %-Ash %-Crude fiber %.

### Animal management

This experiment was conducted from July to September 2020 in a mink farm located at Haiyang City, Yantai, Shandong Province (36°46′12″ N, 120°57′00″ E). All minks were individually housed in standard cages within two-row shelters with bilateral open sides. The minks were fed twice daily (5:00 and 17:00) with ad libitum access to water throughout the experimental period. Routine vaccination procedures against canine distemper virus and parvovirus were administered prior to the trial according to standard mink farming protocols.

### Determination of growth performance

Minks were weighed after a 10-h fast at the start of the trial and at the end of week 4 and week 9 to calculate average daily gain (ADG). The daily feed offered and leftovers were recorded to calculate the average daily feed intake (ADFI) and feed to gain ratio (F/G). At the end of the trial, six minks per group were randomly selected and euthanized. The minks were placed horizontally on a flat surface, and the body length was measured from the tip of the nose to the base of the tail. The pelt was then removed and the fresh pelt weight was determined. The pelt length was measured from the nose tip to the tail base of the harvested mink skin.

### Apparent digestibility of nutrients

During the last 3 days of weeks 4 and weeks 9, feces were collected using the total collection method for digestibility trials. Six minks with similar body weights were selected from each group, and feces were continuously collected from each mink for 3 days. Immediately after collection, the feces were treated daily with a 10% sulfuric acid solution (5% of fresh weight) for nitrogen fixation. For each mink, feces collected over the three-day period were thoroughly mixed, and representative samples were taken and stored at −20°C for further analysis. Meanwhile, feed samples corresponding to the 3-day fecal collection period were homogenized, and representative samples were stored at −20°C until analysis. Both feed and fecal samples were oven-dried at 65°C to constant weight, ground to pass through a 40-mesh sieve, and prepared as air-dried samples for nutrient digestibility analysis. Apparent digestibility was assessed using samples from six minks per group for all nutrients except amino acids. For amino acids, a subset of three fecal samples per group was selected from the original six for analysis. Nutrient contents were determined according to Chinese National Standards: dry matter by GB/T 6435−2014 [13], crude protein by GB/T 6432−2018 [14], crude fat by GB/T 6433−2006 [15], crude ash by GB/T 6438−2007 [16], calcium by GB/T 6436−2018 [17], phosphorus by GB/T 6437−2018 [18] and amino acid by GB/T 18246−2019 [19]. The apparent digestibility of nutrients was calculated using the following formula: Nutrient digestibility (%) = [(Nutrient intake – Fecal nutrient excretion)/ Nutrient intake] × 100.

### Sample collection

At the end of week 9, six minks were randomly selected from each group. To minimize suffering, the minks were initially anesthetized via intravenous injection of pentobarbital sodium in a dose of 40 mg/kg body weight. Blood samples were collected via cardiac puncture and centrifuged at 3000 rpm for 10 min at 4°C. The resulting serum supernatant was aliquoted and stored at −20°C for further analysis. The minks were then euthanized by an intravenous overdose of pentobarbital sodium (100 mg/kg body weight), and liver samples were collected, immediately snap-frozen in liquid nitrogen, and stored at −20°C for subsequent analysis. Duodenal and jejunal segments (2 cm each) were isolated, flushed with 0.9% (w/v) sodium chloride solution to remove luminal contents, and fixed in 10% formaldehyde solution.

### Determination of antioxidant capacity in serum and liver tissues

For liver homogenate preparation, approximately 1 g of tissue was precisely weighed and homogenized in 9 mL of physiological saline on an ice-water bath to obtain a 10% (w/v) homogenate. The homogenate was then centrifuged at 1,581 × g for 10 min at 4°C. The resulting supernatant was collected for the measurement of hepatic antioxidant capacity. The activities of total antioxidant capacity (T-AOC) (A015-3–1), GSH-Px (A005-1–2), total superoxide dismutase (T-SOD) (A001-1–2), and glutathione S-transferase (GSH-ST) (A004-1–1), as well as the levels of malondialdehyde (MDA) (A003-1–2), reduced glutathione (GSH) (A006-2–1) and oxidized glutathione (GSSG) (A061-1–2) in serum and liver were measured using commercial assay kits (Nanjing Jiancheng Bioengineering institute, China) following the manufacturer's protocols.

### Intestinal morphology analysis

The fixed intestinal samples were removed from the formaldehyde solution, trimmed evenly with a scalpel, and placed in a dehydration cassette. The tissue blocks were sequentially immersed in the following solutions: 75% alcohol for 4 h, 85% alcohol for 2 h, 90% alcohol for 2 h, 95% alcohol for 1 h, absolute ethanol I for 30 min, absolute ethanol II for 30 min, alcohol‑benzene for 5–10 min, xylene I for 5–10 min, xylene II for 5–10 min, molten paraffin wax at 65°C I for 1 h, molten paraffin wax at 65°C II for 1 h, and molten paraffin wax at 65°C III for 1 h. After infiltration, the tissue blocks were embedded with paraffin using an embedding machine, solidified on a cooling plate, and then trimmed with a blade. The trimmed blocks were mounted on a microtome and serially sectioned at 5 μm thickness. The resulting sections were floated in a 40°C water bath to spread, transferred onto glass slides, dried at 60°C, and finally subjected to hematoxylin‑eosin staining. The specific procedure for hematoxylin-eosin staining was performed according to the method described by Cardiff et al. [20]. The intestinal sections were examined under a Carl Zeiss optical microscope at 50 × magnification. Ten intact villi and their associated crypts were assessed per intestinal section. Villus height and crypt depth were measured using ToupView software, and the villus height-to-crypt depth ratio (VH/CD) was calculated.

### Statistical analysis

Data were analyzed by one-way ANOVA using SPSS 22.0 (IBM Corp.), followed by Duncan's multiple range test for post hoc comparisons. Results are presented as mean ± standard error of the mean (SEM). Statistical significance was set at *P* < 0.05.

## Results

### Effects of GSH supplementation on growth performance

As shown in Table 2, compared with the control group, the GSH addition groups did not affect the ADFI during weeks 5–8 and weeks 1–9 (*P* > 0.05). Compared with the 100 and 250 mg/kg GSH groups, the 200 mg/kg GSH group significantly increased the ADFI during weeks 5–8 (*P* < 0.05). The ADFI in the 200 mg/kg GSH group was significantly higher than that of the 250 mg/kg GSH group (*P* < 0.05) during weeks 1–9. During weeks 1–9, the 200 mg/kg GSH group showed the highest values for ADG and the lowest values for F/G among all groups, although no significant differences were observed (*P* > 0.05).

**Table 2:** Effects of glutathione (GSH) on growth performance of minks.

Items^a^	GSH supplementation level (mg/kg)						SEM	*P*-value		
	0	50	100	150	200	250		ANOVA	Linear	Quadratic
Weeks 1–4										
ADFI (g)	228.46	234.80	234.97	234.18	239.85	225.74	1.873	0.246	0.983	0.065
ADG (g)	21.50	21.61	22.71	21.91	24.26	20.68	0.394	0.095	0.691	0.127
F/G	10.64	10.95	10.41	10.82	9.98	11.00	0.152	0.329	0.830	0.507
Weeks 5–9										
ADFI (g)	248.07^abc^	250.00^ab^	240.29^bc^	247.55^abc^	257.68^a^	231.65^c^	2.462	0.020	0.259	0.244
ADG (g)	14.69	14.89	13.22	14.73	16.49	13.19	0.409	0.153	0.885	0.681
F/G	17.11	17.64	18.45	16.92	15.77	18.38	0.439	0.484	0.930	0.804
Weeks 1–9										
ADFI (g)	238.26^ab^	242.4^ab^	237.63^ab^	240.86^ab^	248.77^a^	228.70^b^	1.927	0.035	0.483	0.092
ADG (g)	18.10	18.25	17.97	18.32	20.38	16.94	0.354	0.080	0.891	0.270
F/G	13.87	14.30	14.43	13.73	12.88	14.69	0.267	0.386	0.874	0.631
Body length (cm)	44.17	43.50	43.50	43.17	44.67	43.50	0.265	0.629	0.976	0.570
Pelt length (cm)	64.00	65.00	64.67	64.00	67.33	64.50	0.474	0.341	0.364	0.671
Fresh pelt weight (g)	577.50	649.17	620.17	558.83	672.33	640.33	14.980	0.212	0.285	0.875

Means in the same row marked by different superscripts are significantly different (*P* < 0.05).

^a^ADFI, average daily feed intake; ADG, average daily gain; F/G, feed to gain ratio.

### Effects of GSH supplementation on nutrient digestibility

As shown in Table 3, increasing dietary GSH levels linearly increased the crude fat digestibility during weeks 1–4 (*P* < 0.05). The crude fat digestibility in the 150, 200, and 250 mg/kg GSH groups were significantly higher than that of the control group (*P* < 0.05). GSH addition did not significantly affect the digestibility of dry matter, crude protein, crude ash, calcium and phosphorus during either weeks 1–4 or 5–9 (*P* > 0.05).

**Table 3:** Effects of glutathione (GSH) on nutrient digestibility of minks.

Items (%)	GSH supplementation level (mg/kg)						SEM	*P*-value		
	0	50	100	150	200	250		ANOVA	Linear	Quadratic
Week 4										
Dry matter	69.75	71.64	71.27	71.39	71.15	72.16	0.507	0.825	0.321	0.741
Crude fat	88.40^b^	89.71^ab^	89.84^ab^	91.47^a^	92.02^a^	91.84^a^	0.413	0.039	0.001	0.474
Crude protein	82.20	83.34	83.95	83.96	83.98	83.94	0.309	0.472	0.098	0.238
Crude ash	28.84	33.98	37.70	34.93	32.55	36.90	1.229	0.310	0.177	0.295
Phosphorus	32.65	36.26	36.39	37.70	37.90	40.35	1.020	0.345	0.034	0.805
Calcium	39.88	37.33	41.19	41.96	41.64	44.99	1.044	0.435	0.070	0.583
Week 9										
Dry matter	73.95	73.63	75.74	74.76	75.24	74.00	0.350	0.483	0.571	0.169
Crude fat	94.29	93.58	94.29	95.31	94.98	95.05	0.338	0.698	0.217	0.969
Crude protein	82.75	82.87	83.28	83.80	83.80	83.36	0.294	0.894	0.326	0.529
Crude ash	33.87	32.40	40.61	33.66	34.06	35.13	1.031	0.258	0.834	0.412
Phosphorus	47.49	44.51	49.01	52.63	52.98	50.43	1.131	0.232	0.059	0.557
Calcium	46.74	47.12	54.81	52.73	40.13	50.04	1.700	0.239	0.845	0.367

Means in the same row marked by different superscripts are significantly different (*P* < 0.05).

### Effects of GSH supplementation on amino acid digestibility

As shown in Table 4, GSH levels linearly and quadratically increased the digestibility of cysteine (*P* < 0.05). Compared with the control group, the 100–200 mg/kg GSH groups significantly increased the digestibility of cysteine (*P* < 0.05). Additionally, GSH addition significantly affected the digestibility of isoleucine (*P* < 0.05). Increasing GSH levels linearly increased the digestibility of tyrosine and quadratically increased the digestibility of leucine and phenylalanine (*P* < 0.05). Only the 150 mg/kg GSH group exhibited significantly higher digestibility of isoleucine, leucine, tyrosine and phenylalanine compared with the control group (*P* < 0.05). GSH had no significant effect on total amino acid digestibility (*P* > 0.05). As presented in Table 5, dietary GSH supplementation did not significantly affect the digestibility of individual or total amino acids in male minks during weeks 5–9 (*P* > 0.05).

**Table 4:** Effects of glutathione (GSH) on amino acid digestibility of minks during weeks 1-4.

Items (%)	GSH supplementation level (mg/kg)						SEM	*P*-value		
	0	50	100	150	200	250		ANOVA	Linear	Quadratic
Asp	79.35	79.10	79.68	81.84	80.34	78.34	0.522	0.537	0.940	0.178
Thr	79.29	78.27	78.90	81.94	80.03	78.06	0.564	0.411	0.852	0.256
Ser	79.38	79.65	79.39	82.57	80.33	78.79	0.521	0.380	0.829	0.164
Glu	85.96	86.22	86.71	87.86	87.35	86.87	0.302	0.526	0.173	0.287
Gly	77.82	80.78	81.19	82.90	81.95	81.46	0.543	0.103	0.028	0.048
Ala	82.54	84.15	85.47	85.30	85.66	85.55	0.397	0.134	0.018	0.133
Cys	81.53^c^	82.61^bc^	84.42^ab^	86.46^a^	84.56^ab^	83.59^bc^	0.472	0.017	0.021	0.006
Val	84.30	84.28	85.75	87.51	86.70	85.43	0.409	0.112	0.062	0.074
Met	89.08	90.83	91.83	93.62	94.52	91.79	0.688	0.249	0.067	0.139
Ile	80.80^bc^	76.42^c^	83.00^abc^	88.38^a^	86.08^ab^	81.26^bc^	1.170	0.020	0.055	0.069
Leu	86.01^bc^	84.39^c^	87.04^abc^	89.54^a^	88.00^ab^	86.03^bc^	0.502	0.023	0.100	0.044
Tyr	92.24^bc^	91.07^c^	93.29^abc^	95.04^a^	94.29^ab^	92.76^abc^	0.404	0.026	0.041	0.064
Phe	91.16^bc^	90.57^c^	92.18^abc^	93.38^a^	92.96^ab^	91.42^abc^	0.316	0.041	0.077	0.036
Lys	82.14	82.17	81.69	85.05	84.42	83.04	0.487	0.260	0.142	0.468
His	74.38	75.07	76.63	77.01	81.52	78.53	1.053	0.459	0.085	0.784
Arg	90.52	91.27	91.34	92.16	91.82	91.82	0.211	0.292	0.049	0.255
Pro	81.31	83.14	82.92	84.13	83.31	83.29	0.369	0.416	0.146	0.179
Total AA^a^	83.92	84.09	85.17	86.95	86.19	84.94	0.393	0.169	0.091	0.091

Means in the same row marked by different superscripts are significantly different (*P* < 0.05).

^a^AA, amino acids.

**Table 5:** Effects of glutathione (GSH) on amino acid digestibility of minks during weeks 5-9.

Items (%)	GSH supplementation level (mg/kg)						SEM	*P*-value		
	0	50	100	150	200	250		ANOVA	Linear	Quadratic
Asp	81.12	81.22	82.59	82.23	81.83	82.71	0.327	0.675	0.211	0.692
Thr	78.68	78.58	79.63	79.79	78.37	79.64	0.380	0.852	0.625	0.757
Ser	80.31	80.88	82.11	81.92	80.61	81.68	0.361	0.676	0.469	0.391
Glu	89.31	88.90	90.15	89.21	88.69	89.41	0.225	0.580	0.829	0.797
Gly	82.06	82.24	83.99	83.17	83.01	83.75	0.330	0.512	0.174	0.530
Ala	85.33	84.41	86.28	85.63	85.52	86.64	0.329	0.509	0.202	0.771
Cys	84.44	83.60	85.14	86.24	86.11	88.11	0.549	0.221	0.022	0.518
Val	87.21	86.51	87.92	87.66	87.29	88.05	0.293	0.751	0.350	0.975
Met	84.04	81.98	82.65	86.37	88.73	86.53	0.951	0.299	0.072	0.769
Ile	86.47	85.21	86.13	86.06	87.96	87.47	0.410	0.457	0.142	0.417
Leu	88.33	87.44	88.53	88.45	88.80	88.90	0.242	0.632	0.211	0.732
Tyr	89.13	89.27	89.95	91.14	91.23	91.32	0.384	0.343	0.035	0.759
Phe	89.40	89.67	90.69	91.06	91.03	91.20	0.268	0.211	0.020	0.406
Lys	81.55	80.59	82.20	82.19	81.46	82.13	0.403	0.888	0.559	0.905
His	71.86	69.20	75.23	74.47	71.78	72.99	1.676	0.950	0.752	0.723
Arg	89.18	90.14	90.65	90.24	89.97	89.76	0.246	0.708	0.718	0.156
Pro	87.06	86.67	88.10	87.17	85.01	86.20	0.388	0.326	0.205	0.455
Total AA^a^	85.52	85.14	86.48	86.26	85.90	86.49	0.278	0.720	0.278	0.778

Means in the same row marked by different superscripts are significantly different (*P* < 0.05).

^a^AA, amino acids.

### Effects of GSH supplementation on serum and hepatic antioxidant function

According to Table 6, serum T-SOD activity was significantly increased in a linear manner with increasing GSH levels (*P* < 0.05). The serum T-SOD activity in the 150, 200, and 250 mg/kg GSH groups was significantly higher than that in the control, 50 and 100 mg/kg GSH groups (*P* < 0.05). Increasing GSH levels significantly increased GSH-Px activities in a quadratic manner (*P* < 0.05). Relative to the control group, the 150–200 mg/kg GSH groups significantly enhanced serum GSH-Px activity (*P* < 0.05).

**Table 6:** Effects of glutathione (GSH) on serum antioxidant capacity of minks.

Items^a^	GSH supplementation level (mg/kg)						SEM	*P*-value		
	0	50	100	150	200	250		ANOVA	Linear	Quadratic
MDA (nmol/mL)	10.69	10.88	9.29	9.80	10.05	8.97	0.224	0.078	0.017	0.838
T-AOC (U/mL)	9.59	10.09	9.35	9.21	8.04	9.55	0.296	0.535	0.328	0.636
T-SOD (U/mL)	180.24^b^	185.73^b^	187.30^b^	250.13^a^	246.56^a^	224.23^a^	6.280	<0.001	<0.001	0.101
GSH-Px (U/mL)	1109.33^c^	1363.33^abc^	1333.33^abc^	1754.67^a^	1623.47^ab^	1175.56^bc^	71.259	0.048	0.252	0.010
GSH-ST (U/mL)	29.02	37.64	34.23	35.28	42.89	35.41	1.996	0.598	0.262	0.440
GSH (μmol/L)	18.11	15.82	16.62	18.80	17.35	27.75	1.354	0.083	0.050	0.068
GSSG (μmol/L)	4.37	3.99	4.84	3.77	5.36	5.09	0.191	0.075	0.090	0.385
GSH/GSSG	4.16	4.05	3.44	4.76	3.26	5.58	0.267	0.078	0.260	0.139

Means in the same row marked by different superscripts are significantly different (*P* < 0.05).

^a^MDA, malondialdehyde; T-AOC, total antioxidant capacity; T-SOD, total superoxide dismutase; GSH-Px, glutathione peroxidase; GSH-ST, glutathione S-transferase; GSH/GSSG, reduced glutathione to oxidized glutathione ratio.

Table 7 shows that increasing GSH levels quadratically increased T-SOD and GSH-ST activities in the liver (*P* < 0.05). Hepatic T-SOD activity was significantly higher in the 100 and 200 mg/kg GSH groups than in the control group (*P* < 0.05). Compared with the control group, the 50, 100, and 200 mg/kg groups significantly elevated the GSH-ST activity in the liver (*P* < 0.05). Hepatic GSH levels were increased linearly with increasing dietary GSH levels. The 250 mg/kg GSH group exhibited higher hepatic GSH levels than the control group (*P* < 0.05). The hepatic GSH/GSSG ratio was increased linearly and quadratically with increasing GSH levels (*P* < 0.05). Compared with the control group, all GSH-supplemented groups significantly increased the hepatic GSH/GSSG ratio (*P* < 0.05).

**Table 7:** Effects of glutathione (GSH) on hepatic antioxidant capacity of minks.

Items^a^	GSH supplementation level (mg/kg)						SEM	*P*-value		
	0	50	100	150	200	250		ANOVA	Linear	Quadratic
MDA (nmol/mgprot)	1.03	1.13	0.98	0.99	1.21	1.13	0.040	0.493	0.385	0.500
T-AOC (U/mgprot)	0.61	0.63	0.62	0.69	0.61	0.49	0.019	0.062	0.111	0.016
T-SOD (U/mgprot)	421.20^c^	435.27^bc^	509.22^a^	467.62^abc^	481.12^ab^	414.00^c^	8.861	0.004	0.690	<0.001
GSH-Px (U/mgprot)	52.04	58.74	39.17	49.31	52.66	45.79	1.887	0.058	0.292	0.542
GSH-ST (U/mgprot)	21.82^b^	27.83^a^	26.68^a^	25.06^ab^	27.95^a^	24.62^ab^	0.611	0.006	0.216	0.012
GSH (μmol/gprot)	14.05^b^	18.08^ab^	16.39^ab^	16.55^ab^	19.52^ab^	20.57^a^	0.682	0.046	0.006	0.785
GSSG (μmol/gprot)	3.47	3.10	2.32	2.52	3.21	2.87	0.158	0.306	0.435	0.093
GSH/GSSG	4.05^b^	6.83^a^	7.72^a^	6.69^a^	6.36^a^	7.18^a^	0.349	0.024	0.037	0.038

Means in the same row marked by different superscripts are significantly different (*P* < 0.05).

^a^MDA, malondialdehyde; T-AOC, total antioxidant capacity; T-SOD, total superoxide dismutase; GSH-Px, glutathione peroxidase; GSH-ST, glutathione S-transferase; GSH/GSSG, reduced glutathione to oxidized glutathione ratio.

### Effects of GSH supplementation on intestinal morphology

Results of intestinal morphology were shown in Table 8, S1 and S2 Figs. Increasing GSH levels linearly increased the villus height in the duodenum and jejunum of growing male minks (*P* < 0.05). Compared with the control, only the 200 mg/kg GSH group showed significantly higher duodenal and jejunal villus height (*P* < 0.05). No significant effects of dietary GSH supplementation were observed on crypt depth or the villus height-to-crypt depth ratio in either the duodenum or jejunum (*P* > 0.05).

**Table 8:** Effects of glutathione (GSH) on intestinal morphology of minks.

Items^a^	GSH supplementation level (mg/kg)						SEM	*P*-value		
	0	50	100	150	200	250		ANOVA	Linear	Quadratic
Duodenum										
Villus height (μm)	975.52^b^	956.53^b^	942.75^b^	1074.18^ab^	1169.45^a^	990.79^ab^	25.656	0.034	0.084	0.471
Crypt depth (μm)	602.57	524.52	697.37	656.14	635.49	614.85	21.561	0.416	0.460	0.339
VH/CD	1.66	1.83	1.40	1.66	1.87	1.62	0.056	0.175	0.861	0.695
Jejunum										
Villus height (μm)	1060.22^b^	1185.06^ab^	1096.17^b^	1135.18^b^	1361.02^a^	992.92^b^	34.739	0.014	0.711	0.074
Crypt depth (μm)	596.75	688.94	589.65	697.49	658.41	593.12	18.400	0.297	0.996	0.202
VH/CD	1.79	1.75	1.87	1.70	2.08	1.68	0.064	0.442	0.870	0.598

Means in the same row marked by different superscripts are significantly different (*P* < 0.05).

^a^VH/CD, villus height to crypt depth ratio.

## Discussion

Despite the well-established use of GSH in pharmaceutical and food industries owing to its excellent antioxidant properties [21–23], its application as a feed additive in animals, especially in minks, has been explored relatively recently. Liang et al. [8] found that dietary supplementation with 100 mg/kg GSH could mitigate the decrease in ADG and ADFI in weaned piglets injected with diquat. Liu et al. [11] reported that dietary supplementation with 900 mg/kg GSH increased the final weight, specific growth rate and weight gain rate of Chinese mitten crabs. However, Wang et al. [9] observed that the addition of 75–225 mg/kg GSH in the low fish meal diet did not affect the growth performance of shrimp. Our results showed that dietary GSH supplementation did not significantly affect growth performance or pelt quality in growing male minks compared to the control group. Compared to existing literature on minks, direct studies on GSH supplementation are scarce. The values for growth performance and nutrient digestibility observed in the control group of the current study fall within the normal range previously reported in growing male minks [24–26]. This confirms that our husbandry conditions and dietary formulations were appropriate and provided a reliable basis for evaluating the effects of GSH.

GSH deficiency triggers metabolic reprogramming in the liver, affecting lipid, fatty acid, amino acid, and glucose metabolism. This promotes pyruvate and fatty acid oxidation, while suppressing de novo fatty acid synthesis, thereby disrupting lipid homeostasis [27]. The present study showed that 150–250 mg/kg GSH increased crude fat digestibility during weeks 1–4. This increase in fat digestion may be due to the following two reasons. First, GSH likely helped maintain hepatic health and function, thereby supporting adequate bile acid synthesis and secretion for efficient lipid emulsification [28]. Second, the groups with enhanced fat digestibility (particularly the 200 mg/kg group) concurrently exhibited significant increases in villus height in the duodenum and jejunum. The elongation of intestinal villi expands the mucosal surface area, which may facilitate the absorption and digestion of lipids.

There is limited information regarding the impact of GSH on the apparent digestibility of amino acids in minks. To the best of our knowledge, this study is the first to investigate the effect of dietary GSH supplementation on amino acid digestibility in growing male minks. Maintaining smooth and elastic skin and hair depends on proper nutritional support, especially sufficient amino acid supply. Keratin, which is abundant in cysteine, serine and glycine, is the primary protein found in both the epidermis of the skin and in the hair [10]. In this study, dietary GSH supplementation increased the cysteine digestibility, which may promote skin and hair development in minks. In addition, dietary deficiency of tyrosine and phenylalanine can cause black fur to turn reddish in cats, as low tyrosine and phenylalanine levels impair melanin synthesis, while adequate supplementation maintains natural black pigmentation [29,30]. In our study, GSH supplementation increased tyrosine and phenylalanine digestibility, which indicated that GSH may help improve fur coloration in black minks. Endogenous GSH in the intestinal lumen aids in detoxifying exogenous substances present in food and helps maintain the structural and functional integrity of the intestinal epithelium [31]. The increased amino acid digestibility with GSH supplementation may be attributed to enhanced intestinal antioxidant activity and improved morphology. The present results showed that the significant improvements in amino acid digestibility observed in weeks 1–4 were not maintained in weeks 5–9. This temporal pattern is likely explained by the shifting physiological priorities across growth stages. The early phase corresponds to a period of peak anabolic activity, during which amino acid demand for tissue deposition is highest. At this stage, GSH-mediated improvements in antioxidant capacity and intestinal morphology likely facilitated more efficient digestion of limiting amino acids. As growth plateaued later, the metabolic drive for amino acid absorption declined. Consequently, the effect of GSH on apparent digestibility was no longer significant, despite its continued systemic antioxidant activity.

T-AOC and the activities of T-SOD, GSH-Px, and GSH-ST are widely recognized as primary indicators for assessing antioxidant capacity in the body. In this study, dietary supplementation with GSH increased serum GSH-Px activities and hepatic GSH-ST activities, which can be directly attributed to GSH serving as an essential substrate for both enzymes [32,33]. Notably, all GSH-supplemented groups exhibited a significantly elevated hepatic GSH/GSSG ratio, indicating a substantially improved redox reservoir. This enhanced redox status not only supported sustained GSH-Px activity by maintaining sufficient GSH supply but also provided ample substrate for GSH-ST-mediated detoxification, collectively strengthening the antioxidant and detoxification capacity of minks. SOD catalyzes the conversion of superoxide radicals into hydrogen peroxide and molecular oxygen, thereby eliminating ROS from the cytoplasm [34]. SOD and GSH-Px constitute the first antioxidant defense line, and GSH-ST serves as a second line by catalyzing the conjugation of GSH with toxic compounds generated during oxidative stress [35]. In this study, exogenous GSH increased the activity of T-SOD in both serum and liver, which likely resulted from the improved overall redox state and the subsequent synergistic and protective effects of antioxidant enzymes [11,36]. In line with our results, Liang et al. [8] found that dietary supplementation with 50–100 mg/kg GSH alleviated the diquat-induced decline in serum T-AOC, T-SOD and jejunal T-SOD and GSH-Px activities in piglets.

Intestinal morphology plays an important role in nutrient absorption and overall health in animals. Our results demonstrated that 200 mg/kg GSH increased villus height in both the duodenum and jejunum of minks, suggesting enhanced intestinal absorptive capacity. The elongation of intestinal villi may facilitate more efficient nutrient uptake by amplifying the contact interface between the mucosal epithelium and luminal contents [37]. This observed increase in villus height likely reflects improved enterocyte survival and maturation, similar to the crucial role of GSH in supporting cellular development during the pig oocyte maturation [38]. We speculate that the improved redox state, as evidenced by the elevated hepatic GSH/GSSG ratio, may contribute to this intestinal benefit by reducing oxidative damage to the gut epithelium and supporting cellular repair and growth. This result was consistent with previous studies across species. Wang et al. [9] reported that dietary GSH supplementation at 75–225 mg/kg increased jejunal wall thickness and villus height in the jejunum of shrimp fed low level of fish meal diet. Similarly, Mushawwir et al. [39] found that dietary supplementation of 150 mg/kg GSH combined with 100 mg/kg irradiated chitosan improved ileal villus height and reduced apoptotic cells in quails under heat stress.

## Conclusion

Dietary GSH supplementation enhanced crude fat and amino acid digestibility, boosted systemic and hepatic antioxidant capacity, and improved intestinal morphology in growing male minks, with the 200 mg/kg dose identified as optimal. The findings offer valuable physiological insights and a foundation for future studies under challenge conditions or in combination with other additives to optimize practical use.

## Supporting information

S1 Fig Morphological images of the duodenum in minks. A, 0 mg/kg GSH, control. B, 50 mg/kg GSH. C, 100 mg/kg GSH. D, 150 mg/kg GSH. E, 200 mg/kg GSH. F, 250 mg/kg GSH.
(TIF)

S2 Fig Morphological images of the jejunum in minks. A, 0 mg/kg GSH, control. B, 50 mg/kg GSH. C, 100 mg/kg GSH. D, 150 mg/kg GSH. E, 200 mg/kg GSH. F, 250 mg/kg GSH.
(TIF)

S1 Raw Data (XLSX)
